# Supplementary figures and images for: Reassessing the distribution of Burkholderia pseudomallei outside known endemic areas using animal serological screening combined with environmental surveys: The case of Les Saintes (Guadeloupe) and French Guiana
Source: PLoS Negl Trop Dis. 2024 Sep 26;18(9):e0011977. doi: 10.1371/journal.pntd.0011977 (PMC11515966; doi:10.1371/journal.pntd.0011977)

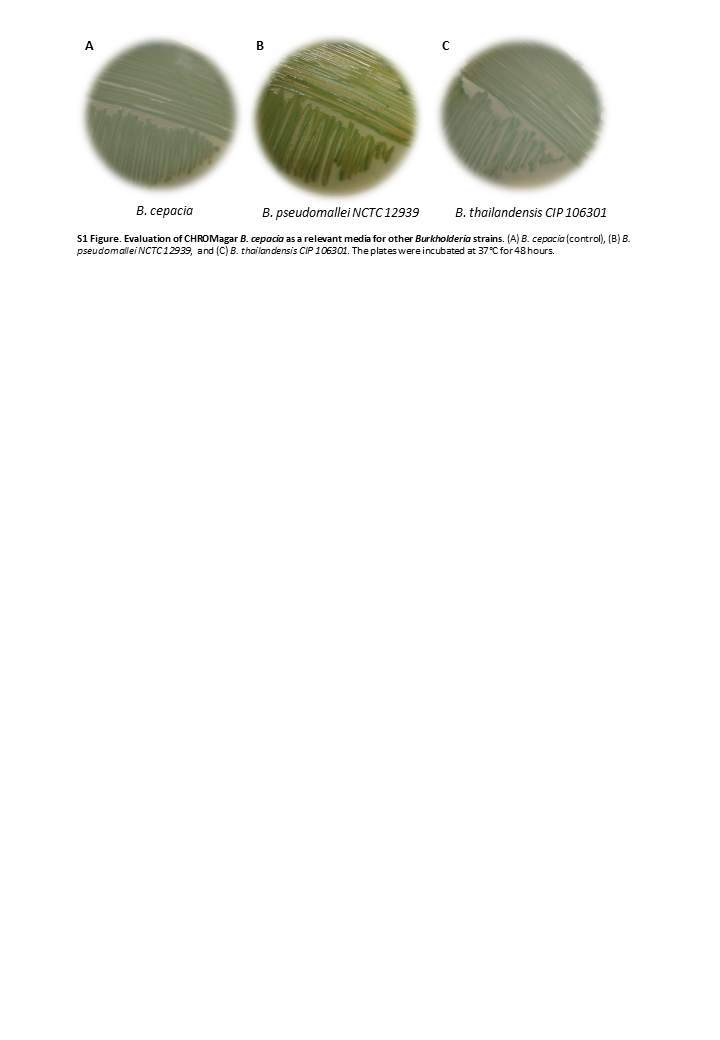

Supplement: S1 Fig — (TIF) [file pntd.0011977.s003.tif]
