# Supplementary material for: Reassessing the distribution of Burkholderia pseudomallei outside known endemic areas using animal serological screening combined with environmental surveys: The case of Les Saintes (Guadeloupe) and French Guiana
Source: PLoS Negl Trop Dis. 2024 Sep 26;18(9):e0011977. doi: 10.1371/journal.pntd.0011977 (PMC11515966; doi:10.1371/journal.pntd.0011977)
Supplement: Attachment — Submitted filename: Replies to reviewers.pdf [file pntd.0011977.s004.pdf]

Dear Professors Carlton and Blacksell,

Please find enclosed our fully revised manuscript: "Reassessing the distribution of *Burkholderia pseudomallei* outside known endemic areas using animal serological screening combined with environmental surveys: the case of Les Saintes (Guadeloupe) and French Guiana", submission number : PNTD-D-24-00204 for publication in your journal.

We would like to thank you, the editorial staff and the reviewers for the helpful comments that have significantly improved the quality and clarity of the manuscript. As required, two versions of the revised manuscript are provided: one with the changes highlighted and a second, clean version. We also provide our point-by-point replies to the reviewers' comments in the below document.

Response to Reviewers file.

The reviewers agreed that the manuscript describes important research on *Burkholderia pseudomallei*. However, they highlight several key concerns that must be addressed on review. First, there is need for greater clarity in the methods - as the current presentation left the reviewers confused about sample collection and analytical methods. Strive to write methods in a way such that they could be replicated by others. Second, there were concerns about ethical approval for the work with animals, and that, at samples may have been collected before approval was obtained. This should be clarified. Third, there is a request for careful editing of the manuscript to make sure the methods, results and interpretation are clear. Additional comments are provided by the reviewers, below.

We thank you for this encouraging comment. Keeping the need for clarity in mind, we have fully revised the methods section. We have also responded to the questions concerning the ethical approvals for our work by adding in a new section at the end of the methods section where the reglementary aspects are more clearly presented.

Reviewer's Responses to Questions

Reviewer #1:

-Are the objectives of the study clearly articulated with a clear testable hypothesis stated? Yes

-Is the study design appropriate to address the stated objectives? Unclear. It is unclear whether the study was designed to perform or not perform environmental sampling, longitudinal study and rectal swab in French Guiana from the start. If the seropositivity was observed in French Guiana, why those studies were not conducted in French Guiana.

The study was designed to use serological analyses to determine whether or not animals in these two French departments had been exposed to *B. pseudomallei*. Following an initial screening, surveys were carried out on two seropositive goat herds in Les Saintes to study the serological response of these animals over time and, in parallel, to look for the presence of the bacterium in their environment.

To clarify this point, the text has been modified as follows: page 4, Line 99. The first objective of this study was to assess the exposure of different livestock species (goats, cattle, horses, sheep) to *B. pseudomallei* in Les Saintes, a group of islands in the Guadeloupe archipelago, and in French Guiana. The second objective was to confirm the local presence of the bacteria on farms with seropositive animals by molecular testing of faecal and environmental samples, and isolation of *B. pseudomallei* by culture.

It is also unclear how positive-control for sera for each species were from? This is very important as the analysis for positivity and S/P% are exclusively based on how positive control for sera from each species are from.
As stated in line 157, for the ELISA and CFT tests, the positive control used is provided in the kit, and its composition is not known.
For the Luminex test, we used serum from a horse naturally infected with *B. mallei*. To clarify this point, the text has been modified as follows: page 7, line 179 "A serum from a horse naturally infected with *B. mallei* (identified as MRI#1) was used as a positive control to determine S/P%, which was calculated for each antigen using the same formula as for the GLANDA ELISA. S/P% values greater than 45% for GroEL and 43% for Hcp1 were considered positive, as previously established". We hope that this is now clearer in the revised version.

-Is the population clearly described and appropriate for the hypothesis being tested? Unclear. The farm selection for the cross-sectional and longitudinal study is unclear. Convenience sampling is not wrong, but clarity and real reasons how those two were selected are needed.

The goat farms in Les Saintes were selected because a high percentage (39%) of seropositivity was detected during the first visit. These farms were also retained for further investigation because of the accessibility of the farms and the interest of the farmers in participating in the study.
To clarify this point, the text was separated into two parts in order to more clearly present the sampling conducted in French Guiana and that conducted in Les Saintes (page 5, line 108 and line 120). We have also added text to clarify the origin of the samples as follows "Blood samples were collected between March 2021 and September 2023 as part of an annual surveillance program conducted by the Direction of Food, agriculture and forests (DAAF - Direction de l'Alimentation, de l'Agriculture et de la Forêt) in French Guiana. A subsample of this serobank was provided by the DAAF veterinarian and included: 361 cattle from 37 farms, 131 sheep from 15 farms, 100 goats from 10 farms, 63 equines from six farms, and 15 pigs from one farm".
We added: "In Les Saintes, on the island of Terre-De-Haut, two goat farms were included in this study and monitored by a local veterinarian: Farm A housed approximately 40 goats and Farm B housed approximately 60 goats. Sampling was not exhaustive as they were free roaming (Fig. 1B). On both farms, mixed-sex goats grazed freely in open pastures, consuming local vegetation, and no birth control measures were used, hence the approximate herd sizes reported by the farmers. The topography of the pastures was characterized by steep terrain and both were susceptible to significant surface water runoff. In November 2021, 31 goats were sampled for blood: 14 from Farm A and 17 from Farm B." (page 5, line 120)

-Is the sample size sufficient to ensure adequate power to address the hypothesis being tested? Unclear. Sample size calculation is not presented.
The aim of this project was to carry out a preliminary study on different farms and in different locations to see if animals could be exposed to *B. pseudomallei* through their environment. No sample size calculations were performed.

- Were correct statistical analysis used to support conclusions? Not relevant. Statistical tests were not used and p values are not presented. This is fine, as descriptive analysis alone in this study is fine.

-Are there concerns about ethical or regulatory requirements being met? No, there are no concerns. However, it is good to present the study approval no. from the relevant EC/IRB.
See page 7, line 139. "Animal sampling protocols have been approved by the IRD Ethics Committee (April 21, 2022). The application submitted to the Ethics Committee covers blood and swab collection by veterinarians on domestic animals in Les Saintes and French Guiana for the period January 1, 2021 to December 31, 2023. Consent was also obtained from the two goat breeders in Les Saintes. In addition,

a Nagoya Protocol dossier (European level, National Decree No. 2017-848 of May 9, 2017) was approved for animal and environmental samplings. Anses is authorized to handle *B. pseudomallei* in accordance with the ANSM regulation on MOT, and to import, analyze and store environmental samples in accordance with the EU Regulation 2019-829 (DAAF, Prefectural Order of 25 August 2022)."

Reviewer #2: (No Response)

Reviewer #3:
-Are the objectives of the study clearly articulated with a clear testable hypothesis stated? Objectives are clearly articulated with a clear hypothesis
-Is the study design appropriate to address the stated objectives? Study design is appropriate, however some methods need some more attention.
Please see the replies to reviewer 1.

- Were correct statistical analysis used to support conclusions? Statistical analysis looks corrects
-Are there concerns about ethical or regulatory requirements being met? It is not sure how ethical clearance was obtained for the animal and soil sampling studies, there is no mention of ethical clearance
Please see the replies to reviewer 1 on this point.

Reviewer #1:
-Does the analysis presented match the analysis plan? Yes.
-Are the results clearly and completely presented? Unclear. It is difficult to read in many parts in both abstract and main text. Result is abstract is unclear whether all parts of the study has been done in two regions (Guadeloupe and French Guiana) similarly or not.
We have fully revised the abstract and the main text keeping clarity in mind. The manuscript has also been carefully re-read by a native English speaker.

-Are the figures (Tables, Images) of sufficient quality for clarity? Table 2 and 4 should move to supplementary Tables. Improve visualization for Table 2 and 4 are recommended, that would be better than providing all raw data as the current Table 2 and 4.
We have now added a more concise version of tables 2 and 4 to the text and moved the more extensive tables to the Supp S3 and S4.

Reviewer #2: (No Response)

Reviewer #3:
-Does the analysis presented match the analysis plan? please see my reviewer comments, some analysis needs more attention
-Are the results clearly and completely presented? Some results needs more attention - see my reviewer comments
-Are the figures (Tables, Images) of sufficient quality for clarity? Figures need more attention are not from sufficient quality/clarity
We have revised the figures as proposed by the reviewer and have clarified the analysis and interpretation. Please see below for more precise details of the changes.

Reviewer #1:
-Are the conclusions supported by the data presented? Yes, However,

(1) the abstract is not clearly written.
As noted above, the abstract has been modified according to the reviewers' suggestions.

(1.1) For example, "the highest prevalence rates were observed ..." , it is unclear of what this prevalence is; Seroprevalence by ELISA? Soil, water, goat rectal swab prevalence, ?
The observed prevalence corresponds to that observed with the GLANDA ELISA test. To clarify this point, the text has been modified as follows: "In French Guiana, 16% (58/361) of cattle, 2% (2/100) of goats, 3% (4/131) of sheep, 24% (15/63) of horses and 0% (0/15) of pigs were seropositive (Table 1). The highest S/P% was observed in cattle, sheep and horses." (page 14, line 325).

(1.2) The abstract is not clearly written that the environmental sampling was not performed in French Guiana - all numerators and denominators of all testing in those two areas (Guadeloupe and French Guiana) for serological tests and environmental sampling should be clearly written.
The abstract has been modified to clarify this point.

(2) The Author summary is not clearly written.
The author summary has been modified to clarify this point.
English in many parts of this manuscript need intensive editing.
The manuscript has been carefully re-read by a native English speaker

(2.1) The key thing about science is that this approach (serological sampling + environmental sampling) should be considered in "new" areas or areas where endemicity of melioidosis is unclear. This method is not meaningful in known melioidosis-endemic areas. This point is unclear in the final sentence/conclusion of the author summary, "thereby helping to identify areas where the pathogen may be present in the environment."
We have revised the last sentence of the abstract accordingly.

-Are the limitations of analysis clearly described? No. The limitation of accuracy, sample size, positive control, bias are not well written.
To clarify the limitations of our study, a specific paragraph has been added to the discussion.

-Do the authors discuss how these data can be helpful to advance our understanding of the topic under study? Yes.

-Is public health relevance addressed? Yes.

Reviewer #2: (No Response)

Reviewer #3:
-Are the conclusions supported by the data presented? The main conclusions are supported by the data presented

-Are the limitations of analysis clearly described? Limitations of the study are not clearly described
To clarify the limitations of our study, a specific paragraph has been added to the discussion.

-Do the authors discuss how these data can be helpful to advance our understanding of the topic under study? Yes.

-Is public health relevance addressed? Yes.

Reviewer #1: (No Response)

Reviewer #2: Considerations for authors:
#63, 64, 65. We aimed to document animal exposure through a serology study, to document their immune response over time, and to investigate the link between seropositive animals and the presence of *B. pseudomallei* in their close environment.
Comment: It is important to draw attention to the interpretation of the serological test. The authors should not state the positivity of the test for *B. pseudomallei* in any part of the work since the ELISA kit was developed for diagnostic of glanders. Seropositivity for Bp is "possible", but not definitive.
We have now added a sentence at the beginning of the results to clarify that a positive ELISA test should be considered as presumptive.

Confusing text, especially in terms of method, due to the mixing of parts of the studies (serology, environment and laboratory study of *Burkholderia pseudomallei*).
Suggestion: organization of the items from the method. For example, serological studies (survey, additional serology) followed by environmental study and sample processing/bacteria characterization. It is also recommended that the result and discussion follow the same sequence.

103. Animal sampling protocols were approved by the IRD Ethics Committee (21 April 2022) # 85. Blood samples were collected between November 2021 and December 2022.
Is this information correct? Were samples collected before ethical approval?
Please see the replies to reviewer 1 on this point.

100. Rectal swabs were collected in duplicate for each animal between April 2022 and 101 December 2022. Inform that the swab collection was parallel to the blood sample collection (in methods).
During each sampling session conducted at the two goat farms located in Les Saintes, rectal swabs were also taken in addition to blood samples.
To clarify this point, the text has been modified as follows: “Regarding the longitudinal study in Les Saintes, after the initial sampling in November 2021 (described above), blood samples, rectal swabs or feces were collected by the local veterinarian from April 2022 to December 2022 on farm B, and from April 2022 to March 2023 on farm A (Fig. 2)”. (Line 195, page 9)

Reviewer #3: (No Response)

Summary and General Comments</br></br> Use this section to provide overall comments, discuss strengths/weaknesses of the study, novelty, significance, general execution and scholarship. You may also include additional comments for the author, including concerns about dual publication, research ethics, or publication ethics. If requesting major revision, please articulate the new experiments that are needed.

Reviewer #1: This study would have some value for the community if it's well written and clear for all parts involved.

Reviewer #2: The manuscript discusses the presence of melioidosis in Guadeloupe and possible occurrence in French Guiana, raising the important debate about the distribution of the disease beyond the areas considered endemic. I also highlight the approach involving the environment and animal studies, fundamental for the complex ecological understanding of melioidosis. The diversity of *Burkholderia* species and the need for attention to the development of more specific diagnostic tests so

that we can have a better understanding of diseases that affect animals such as melioidosis and glanders, was also considered in the study.

Reviewer #3: Reviewer's comments PNTD-D-24-00204 This article aims to investigate the presence of *B. pseudomallei* in animals and environment in French West. The article is important to increase awareness of *B. pseudomallei* outside the known endemic hot spots.
The authors are to be applauded for their efforts to put the melioidosis problem in French West on the map. Indeed, more understanding of the epidemiology is urgently needed because of the high burden and the neglected status of melioidosis. However, the article needs more attention and the English and figures/tables should be further improved.

Major comments:

- Please remove 'definite proof' from the title and change in for example presence etc. As the serological study does not provides definite proof. Be aware that with this title no reader will understand you also performed a study in French Guiana.

We have now revised the title to "Reassessing the distribution of *Burkholderia pseudomallei* outside known endemic areas using animal serological screening combined with environmental surveys: the case of Les Saintes (Guadeloupe) and French Guiana ".

- To me, Figure 2 together with the methods section at line 95-103, in which the authors refer to the serological analyses being explained later, is quite confusing. Also, the Les Saintes farms contain 31 animals (line 88) which contradicts with the numbers in Figure 2 as these are oftentimes higher dan 31. If both farms have 31 animals, please state this. The figure should be self-explanatory.

We apologise for this confusion. The methods section has now been completely revised to take into account the reviewers comments. The two goat farms house around 40 goats in farm A and around sixty in farm B. During the sampling events, we were unable to capture and sample all of the animals as they live semi-freely. This is why the number of goats sampled varies from one collection to another.
To clarify this point, a paragraph was created in material and methods "Regarding the longitudinal study in Les Saintes, after the initial sampling in November 2021 (described above), blood samples, rectal swabs or feces were collected by the local veterinarian from April 2022 to December 2022 on farm B, and from April 2022 to March 2023 on farm A (Fig. 2). Due to the free-range nature of the farms, goat capture was not exhaustive during each field visit. In April 2022, as many animals as possible were captured, sampled and identified with a plastic collar. However, due to the loss of these plastic collars, microchip re-identification was conducted in August 2022. This date corresponds to sampling #1 of the longitudinal study (sampling #2 occurred in December 2022, sampling #3 in March 2023; see Fig. 2). Blood samples were processed identically to the initial visit, and rectal swabs were stored dry at -20°C until PCR analysis." (page 9, line 194)

- Explain how water samples were collected in the paragraph "Environmental sampling in Guadeloupe". This information is absent. For example, how much water was collected, how was it stored, etcetera.

The information was in the text page 11, line 237. For clarity, a paragraph was created in material and methods "Water samples were collected from goat drinking troughs on farms A (n = 2) and B (n = 8). Within two hours of collection, 50 mL of each water sample was filtered sequentially through a 3 µm filter followed by a 0.2 µm filter. All filters were folded in four, preserved in Lysogeny Broth (LB) (10 g peptone, 5 g yeast extract, 10 g NaCl for 1 L)-glycerol 20% and stored at -80°C until analysis. For analysis, the LB-glycerol 20% was discarded and the 0.2 µm filters were aseptically cut in half. One half was placed in 10 mL of liquid Ashdown medium and followed the enrichment protocol described above, while the other half was stored at -80°C for PCR analysis".

- Please elaborate on the ethical approval for the study, how was ethical clearance obtained for the animal and soil sampling studies?

The ethical committee approval obtained from the IRD covers all animal sampling conducted between January 2021 and December 2023. Informed consent from the breeders was obtained, and the collections were subject to authorization via the Nagoya Protocol.
To clarify this point, a paragraph was created in material and methods "Animal sampling protocols have been approved by the IRD Ethics Committee (April 21, 2022). The application submitted to the Ethics Committee covers blood and swab collection by veterinarians on domestic animals in Les Saintes and French Guiana for the period January 1, 2021 to December 31, 2023. Consent was also obtained from the two goat breeders in Les Saintes. In addition, a Nagoya Protocol dossier (European level, National Decree No. 2017-848 of May 9, 2017) was approved for animal and environmental sampling. Anses is authorized to handle *B. pseudomallei* in accordance with the ANSM regulation on MOT, and to import, analyze and store environmental samples in accordance with the EU Regulation 2019-829 (DAAF, Prefectural Order of 25 August 2022)."

- Line 200: why was the supernatant used for PCR and not the heat-killed bacterial pallet?
For the experiment, we only used supernatant because heat lysis allows to break the membranes and liberate DNA from the supernatant.
Moreover, in France, *B. pseudomallei* is a bacterium regulated by the French National Agency for Medicines and Health Products Safety (ANSM) as a Highly Pathogenic Microorganisms and Toxins (HPT) agent. Therefore, it must be handled in a Biosafety Level 3 (BSL-3) facility, and samples require sterility testing prior to their release. In our case, the pellet is utilized to perform sterility testing before the supernatant from the BSL-3 facility is used for PCR analysis.

- Line 205: why were gentamycin and colistin not tested in the antibiotic disc test?
These two antibiotics were tested to confirm that our strain exhibits resistance to colistin and gentamicin, as described in the literature, but were not included as they are not part of the list of antibiotics tested in humans according to the 2024 EUCAST guidelines (European Society of Clinical Microbiology and Infectious Diseases).

To clarify this point, the text has been modified as follows: "The antibiotics tested included: amoxicillin-clavulanate (20-10 µg), meropenem (10 µg), trimethoprim-sulfamethoxazole (1.25-23.75 µg), chloramphenicol (30 µg), imipenem (10 µg), ceftazidime (10 µg), and tetracycline (30 µg) (Bio-Rad, France), as per standard testing of human samples. In addition, we tested susceptibility to colistin (10 µg) and gentamycin (10 µg) (Bio-Rad, France), which were used as selective antibiotics in our culture media". (page 13, line 288). Results for these antibiotics have been added to supplementary table S2.

- Line 212: why was a *B. pseudomallei* strain for example 1026 b or K96 not used as reference control?
We thank the reviewer for this interesting point. We did not use this reference control K93243 as reference control because data for *B. pseudomallei* are indicated in the EUCAST on which we relied for this analysis. Moreover, the French MOT regulation for *B. pseudomallei* requires multiple authorizations to acquire strains.

- Line 223: it would be interesting to use whole genome sequencing on the obtained *B. pseudomallei* strains
The complete sequencing of the strain has been conducted by MinION MK1B system and MiSeq Illumina and deposited in the European Nucleotide Archive (ENA) under accession number ERZ24210644. Additionally, it will be the subject of a genome announcement.

- Line 247: the following sentence is obsolete "The serological results showed heterogeneity both between farms, differentiating 247 positive and negative farms, as well as within positive farms, where both positive and negative animals were detected". You will always have positive and negative findings within farms and within animals so this does not explain heterogeneity in the results. Please remove this sentence.

This sentence has been removed in the revised version page 12, line 247 in old version.

- In Figure 3, several remarks have to be made as the figure is not clear o Please state where the sheep and equids originate from in the legend.
o Panel C is too small.
o The numbers in blue are frequently incorrectly aligned. Also, I am not sure if this should be displayed here. It might be better to mention this together with the farm IDs and place the median S/P rate on top of the error bars.
o In the legend, please first list all the farms in French Guiana and then in Guadeloupe. Also, rearrange the panels in a similar way.
We have now revised Figure 3. Panel C has been enlarged and moved to the end of the figure. The number of animals per farm has been placed in brackets below the farm number. For a best clarity the average S/P% has been removed. The legend has been changed accordingly.

- Table 2 should be rearranged and preferably removed from the main text. Please pay attention to the following:
o Unsure what the added value is of presenting the data per sampled equid. This can also be summarized in the text.
o The distinction between the farms is unclear. Might be better to use column 1 for this.
o Results should not be mentioned in footnotes.
o Tables should be self-explanatory: state why sometimes two results are shown per study ID, such as "P / P" or "ns / N"
We have now revised Table 2. Table 2 has been summarized into a smaller table providing values per farm page 18. The previous table has been transferred to supplementary data.

- Table 3 requires some changes:
o First of all, I was under the impression only 31 animals were present on the Les Saintes farms. However, on December 2022 on Farm A, a total of 39 animals were tested. Please also see my comments regarding Figure 2.
o Please remove the CT values. You either consider a case positive or not.
To clarify this point, modifications have been made as outlined in Figure 2 and on line 126, page 5 of this document. Ct values were removed as requested in Table 3 on pages 19-20.

- Table 4 should be rearranged. I think the data can easily be summarized in a smaller table in which a comparison is made between farm A and B. For example, state the number of males/females, the age with appropriate statistics based on the distribution of the data, and the pooled positivity rates with, for example, a range of the data.
The aim of the table was not to compare the two farms with each other, but to compare animals within the same farm, in order to highlight the fact that some animals remain positive at all collection times, while others remain negative, raising the question of the source of exposure and the duration of immunity. The table on page 20 has been reduced to retain this information and, as noted in the notes, the full table has been transferred to the supplementary data S4.

- Line 332-333: I am not sure how the sample IDs contribute here. Suggest to remove them.
This applies to the whole text.
The identifiers made it possible to distinguish the two soil samples that were positive with the Bpm complex PCR. Of the two samples, only one was able to detect and isolate *B. pseudomallei*; no strains could be detected or isolated from the second. Therefore, we decided to keep the IDs for the soil sample.

- Table 5 should be rearranged. Please pay attention to the following:
o Please remove the CT values. You either consider a case positive or not.

Following comments from reviewers, Ct values have been removed from table 5 page 21.

- The sequence data of strain D22-10884_S313#20 should be uploaded to an online repository. For example, the ENA database.
As mentioned above, the genome of this strain has been deposited in the European Nucleotide Archive (ENA) under accession number ERZ24210644. Additionally, it will be the subject of a genome announcement.

- Please include a phylogenetic tree that compares the MLST profile of your identified strain to the existing *B. pseudomallei* strains, especially from South America.
As asked for, an MLST phylogenetic tree has been included in the article on page 23 line 465. All American strains for which the ST was available in the PubMLST database have been included.

- Please mention the limitations of the study clearly in a separate paragraph and some of the discussion could be condensed. (For example the serological method used in this paper and the cross reactivity to *B. mallei* (and *B. thailandensis*?). What kind of serological methods would the authors preferable use in future studies?
We thank the reviewer for this suggestion, we have now added a section on the limitation of this work to the discussion.

Minor comments:
- Abstract: please note that the reader won't know where Les Saintes is (include Guadeloupe in the conclusion). The Saintes archipelago is a group of 7 islands in the Caribbean Sea. The island of Les Saintes is divided into two communes, Terre-de-Haut and Terre-de-Bas. In order to clarify this point, a sentence was added line 99 page 5 "The first objective of this study was to assess the exposure of different livestock species (goats, cattle, horses, sheep) to *B. pseudomallei* in Les Saintes, a group of islands in the Guadeloupe archipelago, and in French Guiana".

- Line 3: sentence should not start with 'If…'. Please rephrase. The sentence has been reworded starting with "Although".

- Line 4: please split sentence in 2. Recent studies. The sentence has been reworded to split it into two separate sentences as follows: It is endemic in South and Southeast Asia, and northern Australia, causing an estimated 165,000 human cases annually worldwide. Human cases have been reported in the French West Indies (Martinique and Guadeloupe) since the 1990s.

- Line 9: please change the pathogen to B. pseudomallei. The word pathogen has been replaced by "*B. pseudomallei*".

- Line 10: please include serological à combined serological surveys. This has been corrected in the revised version

- Line 11: without clinical signs of melioidosis? This has been corrected in the revised version.

- Line 19: (B): is unnecessary please remove this. This has been corrected in the revised version

- Line 19: please use another word for 'telluric'. This is not commonly used. The word telluric has been replaced by soil-dwelling, most used to describe *B. pseudomallei*.

- Line 10: Change it to *B. pseudomallei* This has been corrected in the revised version.

- Line 27: references 3-5 don't refer to presence of *B. pseudomallei* in Africa. Add of article with human melioidosis description in Africa.

- Line 31: Please change Several risk factors to Risk factors, such as…This has been corrected in the revised version

- Line 37: It is not clear where these refers to. This has been corrected in the revised version page 3 line 66 "The clinical presentation of the disease is highly variable between individuals and species, ranging from chronic forms to acute septicemia".

- Line 51-53: the following sentence is incorrect "Its detection could be harder in nonendemic areas where the bacterium may be present at lower levels". This sentence contradicts itself as a bacterium cannot be isolated from the soil if the bacterium is nonendemic. Please rephrase. The sentence has been reworded to include the reviewers' comments. "Environmental detection of *B. pseudomallei* is challenging and specific culture protocols are required to isolate the bacterium. Detection may be more difficult in areas of low endemicity or emergence, where the bacterium may be present at lower concentrations and/or in limited locations". (Page 4, line 87)

- Line 58: Some of whom had no history of travel to known endemic areas. The word "known" has been added before endemic areas.

- In Figure 1, panel A it is very hard to see the geographical distribution of the sampling sights. Please add an inset of Les Saintes with all these sites distinguishable from each other. The figure has been modified to include a zoom of the island of Les Saintes, making it easier to locate the two goat farms monitored.

- In Figure 2 the resolution appears to be low. Please add a high-quality figure. This has been corrected in the revised version

- Line 14-15 is it 5m or 6m apart? This has been clarified in the revised version page 10 line 221.

- Line 149: explain MRI#1 as the reader will not familiar with this abbreviation The positive control used for Luminex is a horse serum from a horse naturally infected with *B. mallei*, the control is named MRI#1. To clarify this information, the sentence has been modified "A sera from a naturally *B. mallei* infected horse (MRI#1) was used as positive control to determine S/P%, which was calculated for each antigen using the same formula as in the GLANDA-ELISA" (page 8, line 179).

- Line 234: it will not be clear to the reader what you mean with S/P%, please explain. The S/P% corresponds to the percentage of positivity calculated in relation to the positive and negative reference controls. To clarify this point, the sentence has been modified page 14, line 327 "In French Guiana, 16% (58/361) of cattle, 2% (2/100) of goats, 3% (4/131) of sheep, 24% (15/63) of horses and 0% (0/15) of pigs were seropositive (Table 1). The highest S/P% was observed in cattle, sheep and horses ".

- Line 292: what does complex means here? The *B. pseudomallei* complex currently comprises 8 species of *B. pseudomallei*. In 2020, Laroucau *et al.,* designed a real-time PCR to target 5 of these species: *B. pseudomallei, B. mallei, B. thailandensis, B. humptydoensis,* and *B. okhlahomensis*. The gene targeted by this PCR is *aro*A. To clarify this information, the sentence has been modified as follows page 12, line 263" Initial PCR screening was performed using an aroA-based real-time PCR assay targeting the *B. pseudomallei* complex, which includes B*. pseudomallei*, *B. thailandensis*, as well as 6 other *Burkholderia* species".

- Line 293: remove the word "species". This has been corrected in the revised version.

- Line 346: please do not use the following abbreviation CHR_GGF. This has been corrected in the revised version.

- Line 346: suspected colonies? Please remove this whole sentence as this is not of interest. Same applies to sentence 348 Fifteen suspect colonies were isolated from soil. For example, you can start instead with: 'Fifteen colonies were tested PCR-positive for the *B. pseudomallei* complex but ..' etc. Of all the colonies present on the agar plates, only those with a morphology similar to that of *B. pseudomallei* were tested by PCR. To clarify this point, the sentence has been modified in the revised version: page 23, line 451 "As all of the colonies obtained from plating soil 22-10884_313 looked similar, one *B. pseudomallei* colony (22-10884_313#20) from modified CHROMagarTM agar *B. cepacia* was selected for further analysis".

- Line 363: why was only one colony selected for further analysis?
Several colonies present on the agar plates were tested, but since they all had the same antibiogram and genomic sequencing characteristics, only one was selected to be presented in the article.

- Line 381: please explain what you mean with CFT. In the study cited, the authors use CFT (Complement Fixation Test) as their analysis method.

- Line 383: explain what you mean with "within-farm variability».
We wanted to use this term to reflect the fact that seropositive and seronegative animals can be found on the same farm, raising the question of the source of contamination of these animals.
To clarify this point, the text has been modified as follow "Our serological screening in French Guiana and Les Saintes showed that not all farms tested had seropositive animals, regardless of the species tested. This suggests that not all animals are exposed to environmental *B. pseudomallei" page 25 line 498.*

- Line 387-388: the authors mention "within-farm variability" but they give an explanation for between-farm variability "why animals living in farms with similar environmental exposure do not all show the same serological result". Rephrase this so it is correctly explained.
See answer above and following comments from reviewers, the discussion has been revised to clarify its content.

Reviewer #1: No
Reviewer #2: Yes: Dionne B Rolim
Reviewer #3: No
